# Discordances between pediatric and adult thresholds in the diagnosis of hypertension in adolescents with CKD

**DOI:** 10.1007/s00467-021-05166-w

**Published:** 2021-06-25

**Authors:** Elizabeth Black, Jason Lee, Joseph T. Flynn, Charles E. McCulloch, Joshua A. Samuels, Divya Seth, Bradley Warady, Susan Furth, Mark Mitsnefes, Elaine Ku

**Affiliations:** 1 Division of Pediatric Nephrology, Department of Pediatrics, University of California San Francisco, 550 16th street, 5th Floor, San Francisco, CA 94158 USA; 2 Division of Pediatric Nephrology, Department of Pediatrics, University of Michigan, Ann Arbor, MI USA; 3 Division of Pediatric Nephrology, Department of Pediatrics, Seattle Children’s Hospital, Seattle, WA USA; 4 Department of Epidemiology and Biostatistics, University of California San Francisco, San Francisco, CA USA; 5 Division of Pediatric Nephrology, Department of Pediatrics, University of Texas, Houston, TX USA; 6 Division of Nephrology, Department of Medicine, University of California San Francisco, San Francisco, CA USA; 7 Division of Pediatric Nephrology, Department of Pediatrics, Children’s Mercy Hospital, Kansas City, Missouri USA; 8 Division of Pediatric Nephrology, Department of Pediatrics, Children’s Hospital of Philadelphia, Philadelphia, Pennsylvania USA; 9 Division of Pediatric Nephrology, Department of Pediatrics, Cincinnati Children’s Hospital, Cincinnati, OH USA

**Keywords:** Adolescents, Hypertension, Blood pressure, Chronic kidney disease

## Abstract

**Background:**

Adolescents with chronic kidney disease (CKD) are a unique population with a high prevalence of hypertension. Management of hypertension during the transition from adolescence to adulthood can be challenging given differences in normative blood pressure values in adolescents compared with adults.

**Methods:**

In this retrospective analysis of the Chronic Kidney Disease in Children Cohort Study, we compared pediatric versus adult definitions of ambulatory- and clinic-diagnosed hypertension in their ability to discriminate risk for left ventricular hypertrophy (LVH) and kidney failure using logistic and Cox models, respectively.

**Results:**

Overall, among 363 adolescents included for study, the prevalence of systolic hypertension was 27%, 44%, 12%, and 9% based on pediatric ambulatory, adult ambulatory, pediatric clinic, and adult clinic definitions, respectively. All definitions of hypertension were statistically significantly associated with LVH except for the adult ambulatory definition. Presence of ambulatory hypertension was associated with 2.6 times higher odds of LVH using pediatric definitions (95% CI 1.4–5.1) compared to 1.4 times higher odds using adult definitions (95% CI 0.8–3.0). The c-statistics for discrimination of LVH was statistically significantly higher for the pediatric definition of ambulatory hypertension (c=0.61) compared to the adult ambulatory definition (c=0.54), and the Akaike Information Criterion was lower for the pediatric definition. All definitions were associated with progression to kidney failure.

**Conclusion:**

Overall, there was not a substantial difference in pediatric versus adult definitions of hypertension in predicting kidney outcomes, but there was slightly better risk discrimination of the risk of LVH with the pediatric definition of ambulatory hypertension.

**Graphical abstract:**

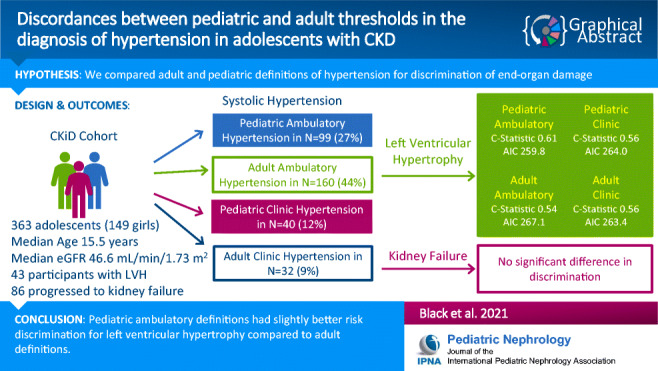

**Supplementary Information:**

The online version contains supplementary material available at 10.1007/s00467-021-05166-w.

## Introduction

In 2017, the American Academy of Pediatrics (AAP) released new clinical practice guidelines for the screening and management of pediatric patients with elevated blood pressure (BP) [1] that included updated definitions of hypertension in children and adolescents, including the adoption of an absolute clinic BP threshold to define hypertension in adolescents ≥13 years old in accordance with adult guidelines [2]. The introduction of these new definitions is likely to increase the prevalence of clinic-diagnosed hypertension in adolescent populations [3, 4], though the benefit of achieving lower BPs on the risk of left ventricular hypertrophy (LVH) and kidney failure is not as strongly supported by evidence based on the development of end-organ damage in younger adults. AAP guidelines for the interpretation of clinic and ambulatory BP readings in children currently rely on the use of normative data based on age, height, and sex. In contrast, adult guidelines have fixed thresholds for the definitions of hypertension that are independent of age, height, and sex [5]. Differences in the normative values used in adult and pediatric definitions of hypertension can lead to diagnostic confusion, particularly as patients transition from adolescence to adulthood, and normative values for children may be higher than the adult hypertension thresholds.

Children and adolescents with chronic kidney disease (CKD) are a unique population in whom these issues are even more important given the high prevalence of hypertension in this population and the role of hypertension in accelerating CKD progression [6]. Our objective in this study was to determine differences in the ability of pediatric versus adult definitions of ambulatory and clinic-diagnosed hypertension to discriminate clinical outcomes in an adolescent population with CKD. Outcomes of interest included LVH and onset of kidney failure. We hypothesized that using pediatric definitions of hypertension would result in better risk discrimination for the development of LVH and kidney failure when compared to use of adult definitions.

## Methods

### Study population

**Fig. 1:**
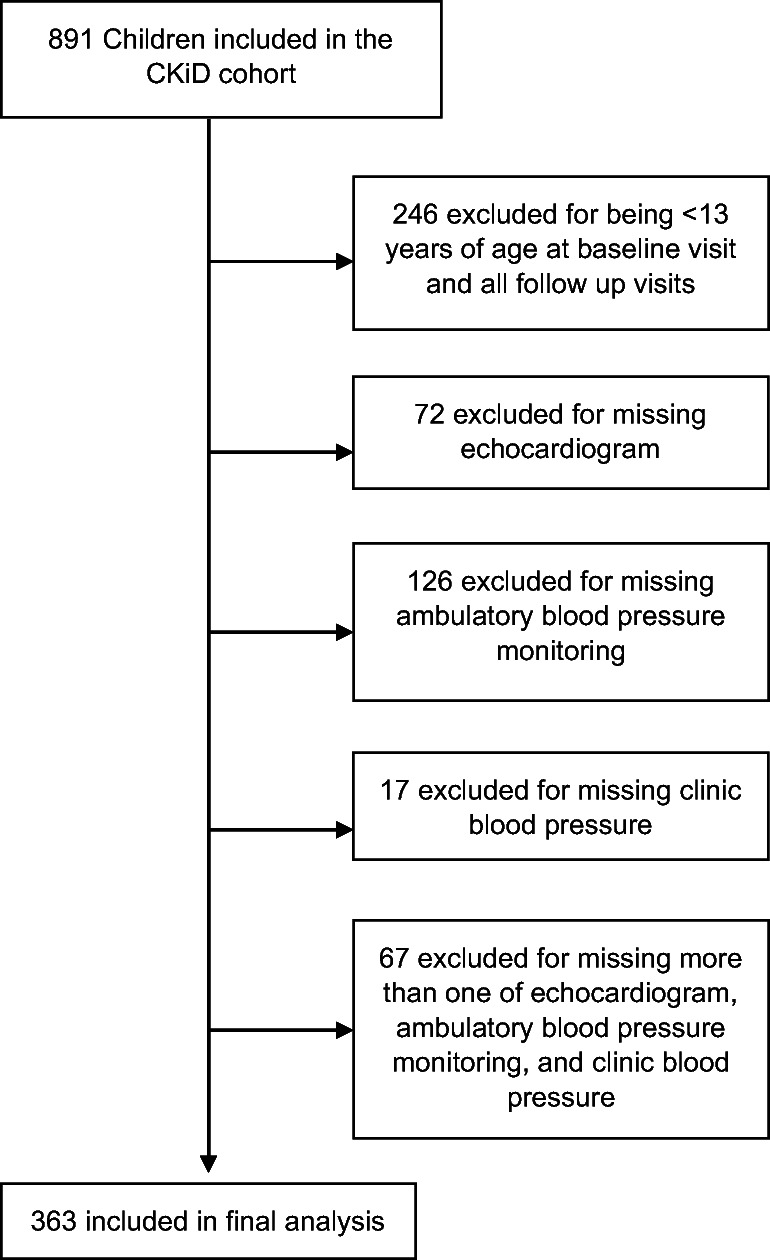
Flow chart of inclusion of patients in study

Details of the Chronic Kidney Disease in Children Cohort Study (CKiD) have been previously described [7]. Briefly, CKiD is an ongoing prospective multicenter cohort study of children between ages 1–16 years with estimated glomerular filtration rate (eGFR) between 30 and 90 mL/min/1.73 m^2^ [7, 8] that aims to determine risk factors for progression of kidney disease and cardiovascular outcomes. CKiD participants were included in our analysis if they had echocardiogram, clinic BP, and ambulatory BP data at any visit where they were at least 13 years old. Of the 891 study participants, 246 were excluded due to age less than 13 years at the baseline or subsequent follow-up visits. An additional 282 individuals were excluded due to either missing echocardiogram (N = 72), ambulatory BP monitoring (N = 126), clinic BP (N = 17), or more than one of these elements (N = 67). A total of 363 adolescents with CKD met the criteria for inclusion in our analysis (Fig. 1).

The CKiD Study protocol has been reviewed by the Institutional Review Boards of each participating center. Informed consent was obtained from study participants across all CKiD sites. De-identified data were obtained from the NIDDK Central Repository. Data collection began October 2003 and were updated through July 2014. The University of California San Francisco Institutional Review Board considers this study not human subjects research. Data that support the findings of this study are available publicly through the National Institute of Diabetes and Digestive and Kidney Disease Central Repository (https://www.niddkrepository.org/home/).

### Predictors of interest

We focused primarily on systolic blood pressure (SBP) to define hypertension and examined its association with adverse outcomes throughout this study given that diastolic norms change minimally with height and age in adolescents [1, 9]. Additionally, prior studies have demonstrated the greater prognostic importance of SBPs, as opposed to diastolic blood pressures (DBPs) in their association with LVH [10–12] and kidney failure [13] in children with CKD. However, we also examined DBP in separate analyses.

#### Ambulatory blood pressure monitoring

Ambulatory BP monitoring was performed during the CKiD Study using a SpaceLabs 90217 monitor (SpaceLabs Healthcare, WA), with BPs taken every 20 min over 24h and centrally analyzed as described previously [12, 14]. Ambulatory BP monitoring was performed at baseline and during follow-up visits (and typically on the same day as the research echocardiogram) every 2 years. We evaluated awake or sleep ambulatory BP readings separately as in prior CKiD studies [11, 14–17].

#### Clinic blood pressure

All clinic-based BPs were performed on the mid-upper arm by trained and certified personnel by auscultation during the CKiD Study annually using an aneroid sphygmomanometer with an appropriate cuff size [1]. Recertification of personnel obtaining BPs and calibration of the aneroid device occurred annually. Three consecutive seated readings were obtained at each study visit 30 s apart after at least 5 min of quiet rest, and the average of these three readings was considered the clinic BP for that visit. The clinic BP from the visit closest in time to ambulatory BP monitoring performance (median time difference between clinic BP and 24-h ambulatory BP monitoring was 0 days; interquartile range, 0–1 days) was used as one of the predictors of interest. This approach is consistent with the methods used in prior CKiD studies [17].

#### Hypertension definitions

We generated dichotomous predictors from both pediatric and adult ambulatory and clinic BP guidelines to define the presence of systolic hypertension and evaluated the strength of the association between pediatric clinic and pediatric ambulatory and adult clinic and adult ambulatory hypertension with each outcome of interest in separate models.

We defined pediatric ambulatory systolic hypertension using SBP norms based on sex and height as in prior CKiD studies [9, 12, 17] and considered an awake SBP ≥ the 95th percentile for sex and height or asleep SBP ≥ the 95th percentile for sex and height to meet the definition of pediatric ambulatory hypertension. We defined adult ambulatory systolic hypertension using systolic BP thresholds from the AHA guidelines (mean awake systolic BP ≥ 130 mmHg or mean sleep SBP ≥ 110 mmHg) [2]. Next, we defined pediatric clinic systolic hypertension as a SBP ≥ 95th percentile for age, sex, and height as per guidelines published in 2004 but used updated normative tables from AAP 2017 guidelines to ensure contemporary relevance of this definition [18]. For pediatric clinic systolic hypertension, we did not incorporate any adult definitions of hypertension as per prior 2004 AAP guidelines*.* Adult clinic systolic hypertension was defined as SBP ≥ 130 mmHg [2].

In sensitivity analysis, to address new changes in the 2017 pediatric clinical practice guidelines where adult thresholds are incorporated into the definition of hypertension for those ≥ 13 but < 18 years of age, we separately defined hypertension using a composite hypertension definition that incorporated either pediatric or adult thresholds. Specifically, the composite ambulatory systolic hypertension definition was defined as awake or sleep SBP ≥ 95th percentile for sex and height or awake SBP ≥ 130 mmHg or sleep SBP ≥ 110 mmHg by ABPM, whichever is lower. The composite clinic systolic hypertension definition was expanded to include either clinic SBP ≥ 95th percentile for age, sex, and height or SBP ≥ 130mmHg, whichever is lower. See Table 1 for a summary of all hypertension definitions used in this analysis.

**Table 1:** Blood pressure definitions

BP metrics	Definition
Systolic hypertension	
Pediatric ambulatory systolic threshold	Awake or sleep systolic BP ≥ 95th percentile for sex and height
Pediatric clinic systolic threshold	Systolic BP ≥ 95th percentile for age, sex, and height
Adult ambulatory systolic threshold	Awake systolic BP of > 130 mmHg or sleep systolic BP > 110 mmHg
Adult clinic systolic threshold	Systolic BP > 130 mmHg
Composite ambulatory threshold	Awake or sleep systolic BP ≥95th percentile for sex and height or awake systolic BP ≥ 130 mmHg or sleep systolic BP ≥ 110 mmHg, whichever is lower
Composite clinic threshold	Systolic BP ≥130 mmHg or ≥95th percentile for age, whichever is lower
Diastolic hypertension	
Pediatric ambulatory diastolic threshold	Awake or sleep diastolic BP ≥ 95th percentile for sex and height
Pediatric clinic diastolic threshold	Diastolic BP ≥ 95th percentile for age, sex, and height
Adult ambulatory diastolic threshold	Awake diastolic BP of > 80 mmHg or sleep diastolic BP > 65 mmHg
Adult clinic diastolic threshold	Diastolic BP > 80 mmHg
Composite ambulatory diastolic threshold	Awake or sleep diastolic BP ≥95th percentile for sex and height or awake diastolic BP ≥ 80 mmHg or sleep diastolic BP ≥ 65 mmHg, whichever is lower
Composite clinic diastolic threshold	Diastolic BP ≥80 mmHg or ≥95th percentile for age, whichever is lower

*BP* blood pressure

For diastolic hypertension, we defined pediatric ambulatory diastolic hypertension using DBP and considered an awake DBP ≥ the 95th percentile for sex and height or asleep DBP ≥ the 95th percentile for sex and height to meet the definition of pediatric ambulatory diastolic hypertension. We defined adult ambulatory diastolic hypertension using DBP thresholds from the AHA guidelines (mean awake DBP ≥ 80 mmHg or mean sleep DBP ≥ 65 mmHg). Next, we defined pediatric clinic diastolic hypertension as a DBP ≥ 95th percentile for age, sex, and height and adult clinic diastolic hypertension as DBP ≥ 80 mmHg. In sensitivity analysis, we defined composite ambulatory diastolic hypertension as awake or sleep DBP ≥ 95th percentile for sex and height or awake DBP ≥ 80 mmHg or sleep DBP ≥ 65 mmHg. Composite clinic diastolic hypertension was defined as either DBP ≥ 95th percentile for age, sex, and height or DBP ≥ 80 mmHg.

### Outcomes of interest

#### Left ventricular hypertrophy

Echocardiograms and 24-h ambulatory BP monitoring were performed every 2 years after the baseline visit. M-mode and Doppler echocardiograms were performed by trained technologists at each CKiD site using a standardized protocol [12]. We defined LVH using the same definitions used in prior CKiD studies, which is a left ventricular mass index ≥ 95th percentile for children and adolescents [12]. The median time difference between 24-h ambulatory BP monitoring and echocardiogram performance was 0 days (interquartile range −1 to 1 days). Our primary analysis of LVH was based on the echocardiogram performed at baseline enrollment or at the first qualifying study visit where participants were ≥13 years of age.

#### Long-term kidney failure ascertainment

Ascertainment of kidney failure onset (defined as the first date of dialysis or transplantation) was performed at annual CKiD visits, by phone follow-up, or by the provision of data from providers. Patients were administratively censored if they were alive as of July 2014 and had not yet developed kidney failure or if they were lost to follow-up at time of the last known study visit.

### Statistical analysis

#### Hypertension and its association with outcomes

First, we used either ambulatory or clinic BP readings to categorize the BP status of each adolescent in the cohort as hypertensive or normotensive by systolic and diastolic definitions and reported the prevalence of HTN according to each of the definitions described above. We compared the baseline characteristics of individuals who were hypertensive by each of these definitions.

Next, we examined the association between BP status (hypertensive or not) based on each of the definitions and LVH using logistic models (cross-sectional analysis) and kidney failure in Cox models (longitudinal analysis) in unadjusted and adjusted analyses. For longitudinal kidney failure analyses, time-to-event was determined starting from the date of the visit when the participant was first ≥ 13 years and had ABPM data available [19]. We considered our primary models unadjusted analyses, since our statistical analysis focuses on the comparison of guideline-recommended definitions of hypertension in their association with outcomes, and in routine clinical practice, other factors are not simultaneously considered when managing hypertension. However, in secondary analysis, we also performed adjusted analysis and accounted for age, sex, race (white, black, or other), body mass index, urine protein-to-creatinine ratio, and estimated glomerular filtration rate as covariates. Results of the adjusted analyses are available in the supplementary materials.

In the sensitivity analysis, we repeated our analyses using the composite ambulatory and clinic hypertension definitions.

#### Risk discrimination using different hypertension definitions

To provide formal tests of the ability of each BP definition to discriminate risk of outcomes, c-statistics were determined for each logistic or Cox model. In logistic models, the c-statistics were determined as the area under the receiver operator curve. In Cox models, Harrell’s c-statistics were used. C-statistics, or concordance statistics, provide a measure of risk discrimination (i.e., the probability that a person with the event has a higher predicted probability than a person without the event). Confidence intervals for c-statistics (to evaluate the discrimination of each Cox model) and their differences (to compare discrimination) were determined via bootstrapping technique (using 500 repetitions). We additionally compared our models using Akaike Information Criterion (AIC). AIC is a numerical expression of the amount of information provided by a model, with a lower number indicating a better fit. An AIC difference of 10 or more is generally considered a significant difference [20]. We repeated our models in adjusted analyses, accounting for the same covariates as described above and determined the c-statistics and AIC for these adjusted models.

Stata 14 (StataCorp, TX: LLC) was used for the performance of all statistical analyses and verified by a separate analyst. P-values <0.05 were considered statistically significant for all analyses.

## Results

**Fig. 2:**
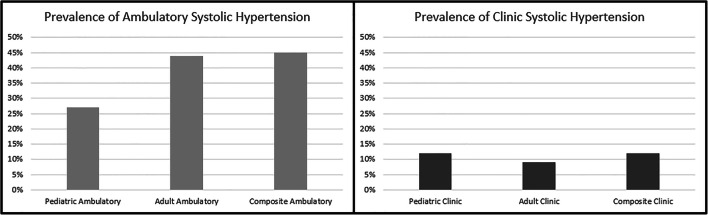
Prevalence of systolic hypertension

Among the 363 adolescents included in this analysis (Fig. 1), the median age was 15.5 years, and 15.2% were black (Table 2). The mean follow-up time was 2.8 years. The prevalence of systolic hypertension was 27%, 12%, 44%, and 9% based on pediatric ambulatory and clinic and adult ambulatory and clinic definitions, respectively. In sensitivity analysis, the prevalence of systolic hypertension was 45% by the composite ambulatory definition and 12% by the composite clinic definition (Fig. 2). The prevalence of diastolic hypertension was 26%, 17%, 31%, and 16% based on pediatric ambulatory and clinic and adult ambulatory and clinic definitions, respectively. In sensitivity analysis, the presence of diastolic hypertension was 31% by the composite ambulatory definition and 18% by the composite clinic definition (Supplemental Figure 1). The characteristics of the overall cohort and those who were found to be hypertensive by each definition are shown in Table 2 for systolic hypertension and Supplemental Table 1 for diastolic hypertension. With regard to the outcomes of interest, 12% of the cohort had LVH, and 24% developed kidney failure over mean follow-up of 2.8 years.

**Table 2:** Baseline characteristics of study by systolic hypertension participants

		Pediatric		Adult		Composite	
Characteristic	Overall	Hypertensive by pediatric ambulatory systolic threshold	Hypertensive by pediatric clinic systolic threshold	Hypertensive by adult ambulatory systolic threshold	Hypertensive by adult clinic systolic threshold	Hypertensive by composite ambulatory systolic threshold	Hypertensive by composite clinic systolic threshold
	N=363	N=99	N=40	N=160	N=32	N=166	N=43
Median age (IQR) in years	15.5 [14.0–17.0]	16.0 [14.0–17.1]	16.0 [14.0–17.1]	16.0 [14.3–17.1]	17.0 [15.0–17.7]	16.0 [14.1–17.1]	16.0 [14.0–17.1]
Female (%)	41.0	44.4	42.5	29.3	37.5	31.3	39.5
Black (%)	15.2	20.2	22.5	19.4	21.9	19.3	20.9
Glomerular cause of CKD (%)	33.1	30.3	30.0	31.3	40.6	30.7	32.6
Use of at least one antihypertensive (%)	72.5	72.7	77.5	68.1	75.0	68.1	76.7
Median eGFR mL/1.73m^2^/minute by Schwartz equation (IQR)	46.6 [29.3–59.8]	38.2 [27.2–56.2]	29.2 [18.5–50.9]	42.5 [25.1–57.4]	32.2 [18.9–53.0]	42.8 [26.2–58.2]	28.9 [18.2–49.7]
Median BMI z-score (IQR)	0.38 [−0.34 to 1.34]	0.30 [−0.50 to 1.22]	0.58 [−0.11 to 1.73]	0.28 [−0.48 to 1.25]	0.78 [0.01–1.86]	0.29 [−0.45 to 1.34]	0.53 [−0.11 to 1.73]
Presence of LVH (%)	11.9	20.2	22.5	13.8	25.0	14.5	20.9
Progression to kidney failure (%)	23.7	30.3	47.5	28.8	46.9	28.3	48.8

*CKD* chronic kidney disease, *eGFR* estimated glomerular filtration rate, *LVH* left ventricular hypertrophy, *BMI* body mass index

The prevalence of masked hypertension was 19% by pediatric systolic hypertension definitions and 35% by adult systolic definitions. The prevalence of white coat hypertension was 3% by pediatric systolic definitions and < 1% by adult systolic definitions. The prevalence of daytime systolic hypertension was 20%, and nocturnal systolic hypertension was 20% by pediatric ambulatory definitions. The prevalence of daytime systolic hypertension was 22%, and nocturnal systolic hypertension was 41% by adult ambulatory definitions.

All definitions of clinic or ambulatory systolic hypertension were statistically significantly associated with odds of LVH except for the adult ambulatory and composite hypertension definitions (Table 3) in unadjusted models. The c-statistic for discrimination of LVH was low across all systolic definitions but statistically significantly higher for the pediatric definition of ambulatory systolic hypertension (c = 0.61) compared to the adult ambulatory systolic definition (c = 0.54) and the composite ambulatory systolic definition (c = 0.56). In sensitivity analysis, we found that the pediatric definition of ambulatory systolic hypertension also had the lowest AIC compared to all other models, though the difference was not large across all definitions of hypertension. There was no statistically significant difference in discrimination for LVH by either the pediatric or adult definitions of clinic systolic hypertension compared to the reference group (Table 3 and Supplemental Table 2).

**Table 3:** Association between different definitions of systolic hypertension and left ventricular hypertrophy (in cross-section) in unadjusted analysis along with model discrimination in adolescents with CKD.

BP metrics N=363	Pediatric		Adult		Composite	
	Pediatric ambulatory systolic threshold	Pediatric clinic systolic threshold	Adult ambulatory systolic threshold	Adult clinic systolic threshold	Composite ambulatory systolic threshold	Composite clinic systolic threshold
Unadjusted OR (95% CI)	2.6 (1.4–5.1)	2.5 (1.1–5.4)	1.4 (0.7–2.6)	2.8 (1.2–6.8)	1.6 (0.8–3.0)	1.2 (0.9–5.0)
Unadjusted c-statistic (95% CI)	0.61 (0.53–0.69)^a^ Reference	0.56 (0.49–0.62)	0.54 (0.46–0.62)*	0.56 (0.49–0.62)	0.56 (0.53–0.69)*	0.55 (0.49–0.61)
Unadjusted AIC	259.8^b^	264.0	267.1	263.4	266.2	264.8
Unadjusted Δ_AIC_^c^	Reference	4.2	7.3	3.6	6.4	5.0

^a^Reference group for c-statistic comparisons

^b^Reference group of AIC comparisons

^c^Δ_AIC_ is the difference in AIC score between the definition of interest and the reference group

*C-statistic was statistically significantly lower compared to reference definition of hypertension

*AIC* Akaike Information Criterion

In unadjusted analysis, the point estimate for the strength of the association between the pediatric ambulatory definition of systolic hypertension and kidney failure was qualitatively not as strong compared to all other definitions, though all definitions were statistically significantly associated with kidney failure. The c-statistic for kidney failure was low across all definitions of systolic or diastolic hypertension using clinic or ambulatory measurements (Table 4 and Supplemental Table 3). There was no statistically significant difference in risk discrimination by c-statistic between any of the definitions of hypertension in unadjusted analysis, but the AIC was lowest for the composite clinic definition when compared to all other definitions of systolic hypertension.

**Table 4:** Association between different definitions of hypertension and kidney failure in unadjusted analysis along with model discrimination in adolescents with CKD.

BP metrics N=363	Pediatric		Adult		Composite	
	Pediatric ambulatory systolic threshold	Pediatric clinic systolic threshold	Adult ambulatory systolic threshold	Adult clinic systolic threshold	Composite ambulatory systolic threshold	Composite clinic systolic threshold
Unadjusted HR (95% CI)	1.7 (1.1–2.6)	2.8 (1.7–4.7)	2.2 (1.4–3.3)	2.4 (1.4–4.2)	2.0 (1.3–3.0)	2.9 (1.8–4.8)
Unadjusted c-statistic 95% CI)	0.55 (0.49–0.61)^a^ Reference	0.57 (0.53–0.62)	0.56 (0.50–0.62)	0.56 (0.51–0.60)	0.56 (0.50–0.62)	0.58 (0.53–0.63)
Unadjusted AIC	881.3^b^	873.0	873.9	878.1	876.4	871.3
Unadjusted Δ_AIC_^c^	Reference	−8.3	−7.4	−3.2	−4.9	−10.0

^a^Reference group for c-statistic comparisons

^b^Reference group of AIC comparisons

^c^Δ_AIC_ is the difference in AIC score between the reference and the AIC score being compared

*AIC* Akaike Information Criterion

With regard to diastolic hypertension, none of the definitions were statistically associated with the outcome of LVH in unadjusted analyses (Supplemental Table 4). Risk discrimination and model fit were similar across all definitions of diastolic hypertension. All definitions of diastolic hypertension were statistically associated with kidney failure, though the c-statistics and AICs were very similar across all definitions (Supplemental Table 5).

## Discussion

In this study of adolescents with CKD, a higher prevalence of hypertension was noted when using 24-h ambulatory monitoring compared to clinic measurements of hypertension. Our data are consistent with prior studies which have shown a prevalence of masked hypertension of 10–38% in children with CKD and reinforces the utility of ABPM in the adolescent CKD population [6, 12, 21, 22].

Overall, we found that the adult clinic definitions of systolic hypertension had the strongest point estimate in terms of its association with LVH in unadjusted analysis, which might have been expected given that the adult SBP threshold of ≥ 130 mmHg used to define hypertension is higher than the 95th percentile of normative values for most adolescents except those who are very tall or close to 18 years in age. In our primary (unadjusted) analysis, we found that the pediatric ambulatory threshold provided better risk discrimination for the outcome of LVH than either the adult or composite ambulatory definitions of hypertension which incorporated pediatric and adult definitions [1]. These findings may reflect challenges in accurately predicting LVH in children, given that the absolute risk discrimination remained low across all definitions, although the pediatric ambulatory definition of hypertension performed better than all other definitions. These findings deserve replication and validation in other cohorts of adolescents with CKD.

For the outcome of kidney failure, in unadjusted analysis, most definitions of systolic hypertension and all definitions of diastolic hypertension were statistically significantly associated with the risk of kidney failure. We did not find any statistically significant differences in risk discrimination provided by any of the definitions of hypertension, although generally clinic-based definitions of hypertension were more strongly associated with kidney failure than ambulatory definitions in unadjusted analysis. The AIC for the composite clinic definition of systolic hypertension was lowest compared to all other definitions. A European randomized control trial previously demonstrated the benefit of intensively lowering mean ambulatory BP to the < 50th percentile for retarding the progression of CKD in children [23, 24]. This benefit is less clear in adults [25, 26] in whom stricter blood pressure control was not found to retard progression of CKD except in those with proteinuria [27] but has been found to reduce the risk of cardiovascular events and mortality [28]. It is important to note that adult trials testing alternative BP targets have mostly enrolled older adults, though a few studies have now specifically examined the young adult age group. One study of younger adults aged 20–40 years in South Korea did find a higher risk of cardiovascular disease outcomes, including myocardial infarction, stroke, and heart failure, among patients with moderate elevations in BP (SBP 120–129, DBP < 80 mmHg) and in those with hypertension [29]. This finding was contradicted by a recent study of patients with CKD aged 21–40 which found no significant difference in the risk of end-organ damage between participants with normal blood pressure and those with moderate elevations in BP (SBP 120–129) [30]. Thus, the applicability of these findings in adolescents remains uncertain.

To our knowledge, no trial data are available to robustly support the appropriate BP targets in the young adult population. Several observational studies have found an increase in the risk of premature death among patients with hypertension in adolescence and young adulthood [31, 32], but the generalizability of these findings to adolescents and young adults with CKD is unclear. One large study of Swedish men found a U-shaped relationship between systolic blood pressure in late adolescence and mortality, with the lowest risk being at 130 mmHg, suggesting that overly aggressive blood pressure control could be associated with harm [33]. Additionally, the risks of long-term antihypertensive therapy over many decades are unknown, since most adults do not begin antihypertensive therapy until middle or older age. Some studies have suggested, for example, that that long-term use of both diuretics [34–37] and beta blockers [38–40] increase the future risk of development of diabetes. Hence, the threshold used to define hypertension in an adolescent or young adult population is important given the need to balance potential side effects of antihypertensive medication with the detrimental effects of elevated blood pressure.

As adolescents with CKD transition into adult healthcare systems, the optimal approach to the diagnosis of hypertension remains unclear. The 2017 AAP guideline chose clinic BP thresholds for adolescents that corresponded to new adult thresholds in order to simplify the identification of hypertension in adolescents and facilitate transition of care. While the 2016 European Society of Hypertension pediatric clinical practice guidelines do recommend adoption of adult ambulatory BP thresholds for older adolescents [41], the current AAP guidelines do not include this recommendation [42]. Additionally, the 2016 European Society of Hypertension clinical practice guidelines use higher thresholds for the diagnosis of adult ambulatory hypertension than the American Heart Association, making comparisons between European and American ambulatory blood pressure data difficult to interpret. Based on our data, using the height-based thresholds for ambulatory systolic hypertension may be acceptable in the adolescent CKD population as it provides modestly better discrimination for LVH when compared to adult and composite definitions of ambulatory hypertension, although the absolute risk discrimination for LVH was overall low.

The strengths of our study include the availability of research-grade clinic and ambulatory BP data from a well-described cohort of children with CKD followed closely for more than a decade with close monitoring for outcomes such as kidney failure and LVH. Limitations include the use of normative pediatric data for ambulatory BP derived from a homogenous European population which may have limited application to the ethnically and racially diverse CKiD cohort. Additionally, only patients with CKD were included in this study, and our findings, including the prevalence of masked and white coat hypertension, may not be applicable to healthy adolescents or adolescents with other chronic conditions. Our study also does not directly address the use of antihypertensive medications, medication adherence, or medication administration timing.

## Conclusion

Hypertension is a common comorbidity in adolescents with CKD. However, it is unclear whether clinicians should use adult or pediatric definitions of hypertension in patients with CKD in this transitional age group. Our findings suggest that the use of pediatric ambulatory definitions of hypertension may be acceptable as these definitions of systolic hypertension had modestly better discrimination for the outcome of LVH among adolescents aged 13 years and older with CKD, although risk discrimination was overall low across all definitions of hypertension. There was no clear difference in predictive ability between adult or pediatric definitions of systolic or diastolic hypertension in terms of the ability to predict kidney outcomes, suggesting that use of either may be acceptable during the transition of care from a kidney perspective. Further confirmation of our findings in other cohorts of children with CKD is needed, and trials testing alternative BP targets in the young adult CKD population are warranted to better inform clinical care of younger populations.

## Supplementary information

ESM 1 (DOCX 29 kb)

ESM 2 (DOCX 27 kb)

## Data Availability

The data used in this research are available through the National Institute of Diabetes and Digestive and Kidney Disease Central Repository (https://www.niddkrepository.org/home/).

## Code availability

Statistical analysis was performed using Stata 14 (StataCorp, TX: LLC).

## Declarations

### **Disclaimer**

This manuscript does not necessarily reflect the opinions or views of the CKiD study, the NIDDK Central Repositories, or the NIDDK.
