# Supplementary figures and images for: Discordances between pediatric and adult thresholds in the diagnosis of hypertension in adolescents with CKD
Source: Pediatr Nephrol. 2021 Jun 25;37(1):179–88. doi: 10.1007/s00467-021-05166-w (PMC8674161; doi:10.1007/s00467-021-05166-w)

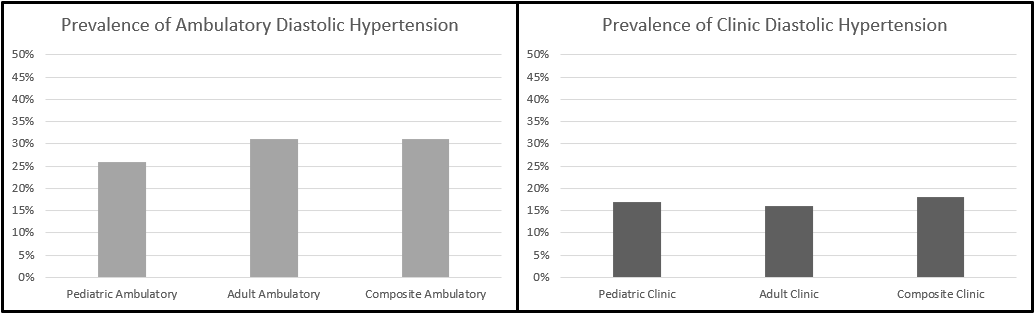

Supplement: Supplementary file 1 — (DOCX 29 kb) [file 467_2021_5166_MOESM1_ESM.docx]
