# Supplementary material for: Discordances between pediatric and adult thresholds in the diagnosis of hypertension in adolescents with CKD
Source: Pediatr Nephrol. 2021 Jun 25;37(1):179–88. doi: 10.1007/s00467-021-05166-w (PMC8674161; doi:10.1007/s00467-021-05166-w)
Supplement: Supplementary file 2 — (DOCX 27 kb) [file 467_2021_5166_MOESM2_ESM.docx]

| **Supplemental Table 1.** Baseline characteristics of study by diastolic hypertension participants. | | | | | | |  | |
| --- | --- | --- | --- | --- | --- | --- | --- | --- |
|  | | Pediatric | | Adult | | Composite | | |
| Characteristic | Overall | Hypertensive by Pediatric Ambulatory Diastolic Threshold | Hypertensive by Pediatric Clinic Diastolic Threshold | Hypertensive by Adult Ambulatory Diastolic Threshold | Hypertensive by Adult Clinic Diastolic Threshold | Hypertensive by Composite Ambulatory Diastolic Threshold | | Hypertensive by Composite Clinic Diastolic Threshold |
|  | N=363 | N=95 | N=62 | N=111 | N=58 | N=111 | | N=65 |
| Median age [IQR] in years | 15.5 [14.0-17.0] | 16.0 [14.1-17.2] | 16.0 [14.0-17.0] | 16.0 [14.1-17.2] | 16.0 [14.1-17.1] | 16.0 [14.1-17.2] | | 16.0 [14.0-17.0] |
| Female (%) | 41.0 | 47.4 | 42.0 | 44.1 | 44.8 | 44.1 | | 41.5 |
| Black (%) | 15.2 | 19.0 | 19.4 | 20.7 | 22.4 | 20.7 | | 20.0 |
| Glomerular cause of CKD (%) | 33.1 | 39.0 | 27.4 | 36.0 | 29.3 | 36.0 | | 29.2 |
| Use of at least one anti-hypertensive (%) | 72.5 | 72.6 | 76.1 | 68.5 | 69.0 | 68.5 | | 67.7 |
| Median eGFR mL/1.73m^2^/minute by Schwartz equation [IQR] | 46.6 [29.3-59.8] | 37.6 [13.3-59.1] | 33.1 [24.8-50.6] | 40.1 [26.9-59.4] | 31.1 [24.7-47.1] | 40.1 [26.9-59.4] | | 33.4 [24.8-50.6] |
| Median BMI z-score [IQR] | 0.38 [-0.34-1.34] | 0.13 [-0.53-0.78] | 0.25 [-0.33-0.86] | 0.18 [-0.52-0.87] | 0.34 [-0.33-0.92] | 0.18 [-0.53-0.87] | | 0.34 [-0.28-0.88] |
| Presence of LVH (%) | 11.9 | 14.7 | 14.5 | 13.5 | 15.5 | 13.5 | | 13.9 |
| Progression to kidney failure (%) | 23.7 | 30.5 | 33.9 | 28.8 | 37.9 | 28.8 | | 33.9 |
| CKD chronic kidney disease; eGFR estimated glomerular filtration rate; LVH left ventricular hypertrophy; BMI body mass index | | | | | | | | |

**Supplemental Table 2.** Association between different definitions of systolic hypertension and left ventricular hypertrophy (in cross-section) in adjusted^a^ analysis along with model discrimination in adolescents with CKD.

| **BP Metrics**  N=363 | **Pediatric** | | **Adult** | | **Composite** | | | |
| --- | --- | --- | --- | --- | --- | --- | --- | --- |
|  | Pediatric Ambulatory Systolic Threshold | Pediatric Clinic Systolic Threshold | Adult Ambulatory Systolic Threshold | Adult Clinic Systolic Threshold | | Composite Ambulatory Systolic Threshold | | Composite Clinic Systolic Threshold |
| **Adjusted OR**^a^  (95% CI) | 1.9 (0.9 – 4.1) | 0.9 (0.3 – 2.5) | 1.3 (0.6 – 2.7) | 1.3 (0.4 – 3.7) | 1.4 (0.7 – 2.9) | | 0.9 (0.3 – 2.4) | |
| **Adjusted C-statistic**^a^  (95% CI) | 0.77 (0.68 – 0.85)^b^ Reference | 0.76 (0.68 – 0.84) | 0.76 (0.68 – 0.84) | 0.76 (0.68 – 0.85) | 0.65 (0.60 – 0.76) | | 0.65 (0.57 – 0.74) | |
| **Adjusted AIC**^a^ | 221.6^b^ | 224.5 | 224.1 | 224.3 | 223.7 | | 224.5 | |
| **Adjusted Δ­_AIC_**^c^ | Reference | 2.9 | 2.5 | 2.7 | 2.1 | | 2.9 | |

^a^Adjusted for age, sex, race, BMI, urine protein to creatinine, and eGFR, N=342 included due to missing covariates

^b^Reference group

^c^Δ­_AIC_ is the difference in AIC score between the definition of interest and the reference group

*C-statistic was statistically significantly lower compared to reference definition of hypertension

AIC Akaike Information Criterion

**Supplemental Table 3.** Association between different definitions of systolic hypertension and kidney failure in adjusted^a^ analysis along with model discrimination in adolescents with CKD.

| **BP Metrics**  N=363 | **Pediatric** | | **Adult** | | **Composite** | | | |
| --- | --- | --- | --- | --- | --- | --- | --- | --- |
|  | Pediatric Ambulatory Systolic Threshold | Pediatric Clinic Systolic Threshold | Adult Ambulatory Systolic Threshold | Adult Clinic Systolic Threshold | | Composite Ambulatory Systolic Threshold | | Composite Clinic Systolic Threshold |
| **Adjusted OR**^a^  (95% CI) | 1.3 (0.8 – 2.0) | 1.6 (0.9 – 2.8) | 1.5 (1.0 – 2.5) | 1.5 (0.8 – 2.8) | 1.5 (0.9 – 2.3) | | 1.4 (0.8 – 2.5) | |
| **Adjusted C-statistic**^a^  (95% CI) | 0.86 (0.82 – 0.90)^b^  Reference | 0.86 (0.82 – 0.90) | 0.85 (0.83 – 0.89) | 0.86 (0.81 – 0.90) | 0.85 (0.82 – 0.90) | | 0.86 (0.82 – 0.90) | |
| **Adjusted AIC**^a^ | 708.6^b^ | 707.3 | 706.1 | 708.1 | 706.8 | | 708.1 | |
| **Adjusted Δ­_AIC_**^c^ | Reference | -1.3 | -2.5 | -0.5 | -1.8 | | -0.5 | |

^a^Adjusted for age, sex, race, BMI, urine protein to creatinine, and eGFR, N=342 included due to missing covariates

^b^Reference group

^c^Δ­_AIC_ is the difference in AIC score between the definition of interest and the reference group

*C-statistic was statistically significantly lower compared to reference definition of hypertension

AIC Akaike Information Criterion

**Supplemental Table 4.** Association between different definitions of diastolic hypertension and left ventricular hypertrophy (in cross-section) in unadjusted and adjusted^a^ analyses along with model discrimination in adolescents with CKD.

| **BP Metrics**  N=363 | **Pediatric** | | **Adult** | | **Composite** | |
| --- | --- | --- | --- | --- | --- | --- |
|  | Pediatric Ambulatory Diastolic Threshold | Pediatric Clinic Diastolic Threshold | Adult Ambulatory Diastolic Threshold | Adult Clinic Diastolic Threshold | Composite Ambulatory Diastolic Threshold | Composite Clinic Diastolic Threshold |
| **Unadjusted OR**  (95% CI) | 1.4 (0.7 – 2.8) | 1.3 (0.6 – 2.9) | 1.3 (0.6 – 2.4) | 1.5 (0.7 – 3.2) | 1.3 (0.6 – 2.4) | 1.2 (0.6 – 2.7) |
| **Unadjusted C-statistic**  (95% CI) | 0.54 (0.46 – 0.61)^b^ Reference | 0.52 (0.46 – 0.59) | 0.52 (0.45 – 0.60) | 0.53 (0.46 – 0.59) | 0.52 (0.45 – 0.60) | 0.52 (0.45 – 0.58) |
| **Unadjusted AIC** | 267.2^c^ | 267.7 | 267.7 | 267.3 | 267.7 | 267.9 |
| **Unadjusted Δ­_AIC_**^d^ | Reference | 0.5 | 0.5 | 0.1 | 0.5 | 0.7 |
| **Adjusted OR**^a^  (95% CI) | 1.1 (0.5 – 2.6) | 0.9 (0.3 – 2.4) | 1.0 (0.4 – 2.2) | 0.9 (0.3 – 2.4) | 1.0 (0.4 – 2.2) | 0.8 (0.3 – 2.2) |
| **Adjusted C-statistic^a^**  (95% CI) | 0.76 (0.68 – 0.84)^b^ Reference | 0.76 (0.68 – 0.84) | 0.76 (0.68 – 0.84) | 0.76 (0.68 – 0.84) | 0.76 (0.68 – 0.84) | 0.76 (0.68 – 0.84) |
| **Adjusted AIC**^a^ | 224.5^c^ | 224.5 | 224.5 | 224.5 | 224.5 | 224.4 |
| **Adjusted Δ­_AIC_**^d^ | Reference | 0 | 0 | 0 | 0 | -0.1 |

^a^Adjusted for age, sex, race, BMI, urine protein to creatinine, and eGFR, N=342 included due to missing covariates

^b^Reference group for c-statistic comparisons.

^c^Reference group of AIC comparisons

^d^Δ­_AIC_ is the difference in AIC score between the reference and the AIC score being compared

*C-statistic was statistically significantly lower compared to reference definition of hypertension

AIC Akaike Information Criterion

**Supplemental Table 5.** Association between different definitions of diastolic hypertension and kidney failure in unadjusted and adjusted^a^ analyses along with model discrimination in adolescents with CKD.

| **BP Metrics**  N=363 | **Pediatric** | | **Adult** | | **Composite** | |
| --- | --- | --- | --- | --- | --- | --- |
|  | Pediatric Ambulatory Diastolic Threshold | Pediatric Clinic Diastolic Threshold | Adult Ambulatory Diastolic Threshold | Adult Clinic Diastolic Threshold | Composite Ambulatory Diastolic Threshold | Composite Clinic Diastolic Threshold |
| **Unadjusted HR**  (95% CI) | 1.9 (1.2 – 3.0) | 1.7 (1.0 – 2.8) | 1.7 (1.1 – 2.7) | 2.3 (1.4 – 3.7) | 1.7 (1.1 – 2.7) | 1.7 (1.1 – 2.8) |
| **Unadjusted C-statistic**  (95% CI) | 0.55 (0.49 – 0.61)^b^  Reference | 0.55 (0.50 – 0.60) | 0.54 (0.49 – 0.60) | 0.56 (0.51 – 0.61) | 0.54 (0.49 – 0.60) | 0.55 (0.50 – 0.60) |
| **Unadjusted AIC** | 878.8^c^ | 881.9 | 880.3 | 876.7 | 880.3 | 881.4 |
| **Unadjusted Δ­_AIC_**^d^ | Reference | 3.1 | 1.5 | -2.1 | 1.5 | 2.6 |
| **Adjusted HR**^a^  (95% CI) | 1.5 (1.0 – 2.5) | 1.0 (0.6 –1.8) | 1.4 (0.9 – 2.3) | 1.2 (0.7 – 2.1) | 1.4 (0.9 – 2.3) | 1.0 (0.6 – 1.8) |
| **Adjusted C-statistic**^a^  (95% CI) | 0.86 (0.82 – 0.90)^b^  Reference | 0.86 (0.82 – 0.89) | 0.86 (0.82 – 0.90) | 0.86 (0.82 – 0.90) | 0.86 (0.82 – 0.89) | 0.86 (0.82 – 0.89) |
| **Adjusted AIC**^a^ | 706.5^c^ | 709.4 | 707.4 | 708.9 | 707.4 | 709.4 |
| **Adjusted Δ­_AIC_**^d^ | Reference | 2.9 | 0.9 | 2.4 | 0.9 | 2.9 |

^­a^Adjusted for age, sex, race, BMI, urine protein to creatinine, and eGFR, N=342 included due to missing covariates

^b^Reference group for c-statistic comparisons.

^c^Reference group of AIC comparisons

^d^Δ­_AIC_ is the difference in AIC score between the reference and the AIC score being compared

*C-statistic was statistically significantly lower compared to reference definition of hypertension

AIC Akaike Information Criterion
